# Rhegmatogenous Retinal Detachment with Giant Retinal Tear: Case Series and Literature Review

**DOI:** 10.3390/jcm13164690

**Published:** 2024-08-09

**Authors:** Siyun Lee, Joonhyung Kim

**Affiliations:** Department of Ophthalmology, CHA Bundang Medical Center, CHA University School of Medicine, #59 Yatap-ro, Bundang-gu, Seongnam 13496, Republic of Korea

**Keywords:** giant retinal tear, pars plana vitrectomy, rhegmatogenous retinal detachment

## Abstract

**Background/Objectives**: This paper reviews three cases of rhegmatogenous retinal detachment (RRD) with giant retinal tear (GRT), focusing on surgical management and outcomes, and synthesizes the current literature on the subject. **Methods**: We retrospectively analyzed three cases of male patients diagnosed with RRD with GRT at our hospital from April 2022 to November 2023. The patients, aged 57, 66, and 60, underwent surgical interventions, with postoperative follow-up extending up to six months. All patients underwent pars plana vitrectomy (PPV), endolaser photocoagulation, fluid–air exchange, and intravitreal gas injection (perfluoropropane (C3F8) 14%) in each case. Surgeries were performed within four days of the patients’ initial visits. Outcome measures included visual acuity improvement, intraocular pressure stabilization, and retinal reattachment rates. Complications in the patients were also evaluated. **Results**: The patient who had not previously undergone cataract surgery (Case 1) had it concurrently with the retinal surgery. Four months after the initial surgery, Case 3 developed a secondary epiretinal membrane (ERM) and subsequently underwent ERM removal surgery. Postoperative care involved the tailored use of anti-inflammatory medications and frequent monitoring to mitigate complications such as proliferative vitreoretinopathy, epiretinal membrane formation, and redetachment. **Conclusions**: While limited by the small sample size, this report underscores the potential benefits of prompt surgical intervention, meticulous postoperative care, and proactive management of complications in RRD with GRT. Insights from these cases, supported by multiple literature reviews, may inform treatment strategies and highlight areas for further research in larger, more diverse patient populations.

## 1. Introduction

Rhegmatogenous retinal detachment (RRD) with giant retinal tear (GRT) is one of the most severe ophthalmologic conditions, often leading to poor visual outcomes. Many patients with RRD with GRT experience significant vision loss, which may not improve substantially and could even result in blindness. This condition can dramatically affect a patient’s quality of life, making it a critical emergency that requires prompt and proper surgical intervention. Proper treatment, especially surgical treatment at the right time, is essential for providing the most benefit and improving outcomes for patients.

GRT is defined as a full-thickness tear of the neurosensory retina extending circumferentially for at least three clock hours (90 degrees or more) in the presence of posterior vitreous detachment (PVD) [1,2,3]. GRTs account for approximately 1.5% of all RRDs [4]. The average age of occurrence is approximately 42 years [2,4]. Generally, there is a greater prevalence among males compared to females, with males representing 72% of all cases [2].

The pathogenesis of GRT involves the liquefaction of the central vitreous and traction at the vitreous base, resulting in a circumferential tear in the neurosensory retina. In GRT, the vitreous gel remains attached to the anterior flap of the retina, while the posterior retina moves freely and can fold. This mobility distinguishes GRT from retinal dialyses, where the vitreous adheres to the posterior tear, limiting the retina’s mobility [5].

Approximately 54% of GRTs have an idiopathic origin, 12.3% are associated with trauma, 25% are due to high myopia, and 14% are linked to hereditary vitreoretinopathies [1,2,5]. Cases of GRT can vary significantly, as demonstrated by recent reports of intermediate periphery tears in spherically large eyeballs [6], unique post-traumatic presentations [7], and specific conditions such as Marfan’s syndrome [8].

One prominent clinical characteristic of GRT is visual symptoms. Patients might experience a sudden appearance of floaters and/or flashes of light, decreased visual acuity, and the presence of a shadow or curtain-like effect across the visual field may indicate retinal detachment (RD) [2,4]. Roughly two-thirds of GRT patients present within 10 days of experiencing visual symptoms [9], while the remaining one-third present later [10]. Vitreous hemorrhage may occur if the tear involves a retinal blood vessel [11]. Numerous GRTs are accompanied by RRD, with approximately half of these cases being macula-off RD [2].

The optimal treatment is pars plana vitrectomy (PPV), involving the correction of the inverted central retinal flap with perfluorocarbon liquids, sealing the retina with endolaser or cryocoagulation, and using silicone oil or gas for retinal tamponade [9,10,12,13,14,15,16,17]. Primary and final rates of retinal reattachment are successful in 88% and 95%, respectively, of GRT patients [2]. However, even with successful retinal attachment, visual improvement might be limited.

This study reviews three cases of RRD with GRT treated at our center and aims to highlight the effectiveness of surgical interventions, evaluate postoperative recovery, and identify factors contributing to successful retinal reattachment as well as visual acuity improvement. Additionally, this study seeks to compare these findings with existing literature to provide insights into optimal management strategies for RRD with GRTs.

## 2. Materials and Methods

We retrospectively reviewed the medical records of 3 patients diagnosed with RRD associated with GRT between April 2022 and November 2023 at CHA Bundang Medical Center (Seongnam, Republic of Korea). The inclusion criteria were patients diagnosed with RRD with GRT at our hospital and who underwent surgical treatment. All three patients received both diagnostic and surgical treatment at our ophthalmology department. The same ophthalmologist diagnosed and operated on all three patients. Ophthalmologically, they underwent vision and intraocular pressure assessments, fundus photography (FP), optical coherence tomography (OCT), and B-scan ultrasound (B-scan). Postoperatively, their ophthalmologic progress was continuously monitored until 6 months, observing changes in vision, intraocular pressure, and fundus condition.

Medical data included patient age, sex, and laterality of GRT. Visual acuities, intraocular pressure measures with a non-contact tonometer (NCT), and findings from slit-lamp examination at presentation and for each subsequent visit were noted.

## 3. Results

- Case 1

**Figure 1:**
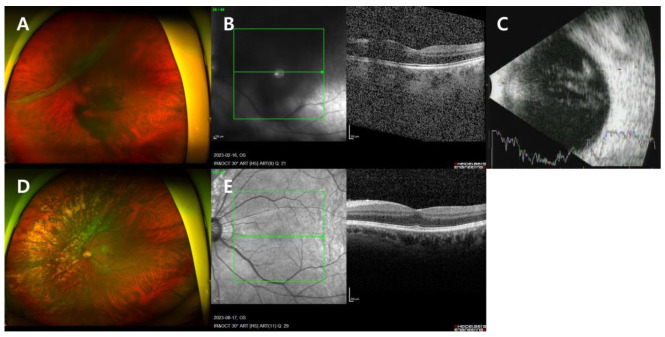
Case 1 patient’s left eye images. (**A**–**C**) On the day of presentation. (**A**) Color fundus photography (FP) showed rhegmatogenous retinal detachment (RRD) with giant retinal tear (GRT) at a 9–12 o’clock direction. (**B**) Optical coherence tomography (OCT) showed vitreous hemorrhage. (**C**) B-scan ultrasound (B-scan) showed vitreous hemorrhage and retinal detachment. (**D**,**E**) Six months after surgery. (**D**) FP showed a flat, well-attached retina. (**E**) OCT showed a flat, well-attached retina.

A 57-year-old male presented with sudden visual acuity decline and floaters in the left eye that began 5 days prior to the visit. The patient arrived with a referral from a local ophthalmology clinic indicating vitreous hemorrhage of the left eye. He had no previous ophthalmic history or other medical conditions. Initial examination showed uncorrected visual acuity of 20/200 in the right eye and a 2 m finger count in the left, with corrected visual acuity of 20/20 in the right eye and 20/100 in the left. Anterior segment examination revealed no significant findings. Fundus photography (FP), optical coherence tomography (OCT), and fundus examination identified multiple atrophic holes in the right eye, vitreous hemorrhage, multiple retinal tears, and GRT from a 9-to-12 o’clock direction associated with RRD in the left eye (Figure 1A–C). The axial length of the left eye was measured at 27.52 mm.

Two days after the visit, the patient underwent barrier photocoagulation (PC) for the atrophic holes in the right eye. Four days later, he received PPV, endolaser photocoagulation, fluid–air exchange, intravitreal gas injection (perfluoropropane (C3F8) 14%), and cataract surgery for the left eye. The next day, postoperative observation revealed a visual acuity of hand motion level in the left eye, an intraocular pressure (IOP) of 12 mmHg, and a well-attached, flat retina. The patient maintained a prone position for 3 days post-surgery, followed by a left decubitus position. Subsequently, the patient underwent three additional barrier PCs in the left eye at 1.5 months, 2 months, and 2.5 months postoperatively.

At the 6-month follow-up, the retina remained flatly attached without any complications, and the patient achieved a best-corrected visual acuity (BCVA) of 20/20 (Figure 1D,E).

- Case 2

**Figure 2:**
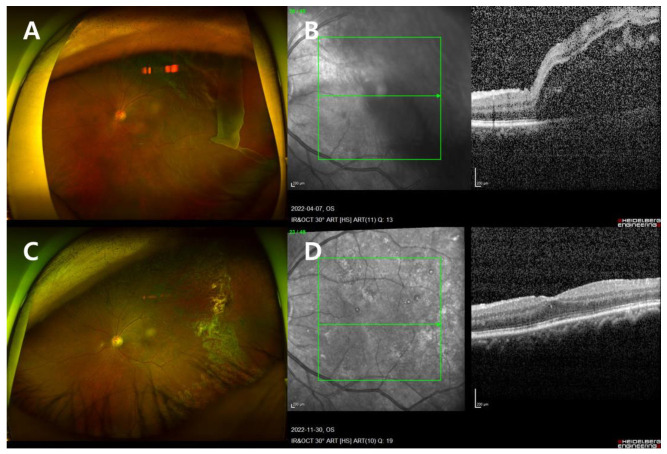
Case 2 patient’s left eye images (**A**,**B**). On the day of presentation. (**A**) FP showed RRD with GRT at the temporal area. (**B**) OCT showed macula-off retinal detachment (RD). (**C**,**D**) Six months after surgery. (**C**) FP showed a flat, well-attached retina. (**D**) OCT showed a flat, well-attached retina.

A 66-year-old man presented with a complaint of the left visual field being partially obscured since the day before his visit. He was referred to our clinic with a diagnosis of retinal detachment in the left eye, from a local ophthalmology clinic. He had a history of cataract surgery in the left eye three years prior, as well as hypertension, benign prostatic hyperplasia, and hyperlipidemia. Upon examination, his uncorrected visual acuity was 20/25 in the right eye and 20/63 in the left eye, with a slit lamp examination revealing 1+ cells in the anterior chamber. FP, OCT, B-scan, and fundus examination confirmed the presence of GRT in the temporal area of the left eye with macula-off RRD (Figure 2A,B). The axial length of the left eye was measured at 23.91 mm.

The patient underwent PPV, endolaser photocoagulation, fluid–air exchange, and intravitreal gas injection (C3F8 14%) in the left eye on the day of his visit. The day after surgery, his visual acuity in the left eye was hand motion, IOP was 12 mmHg, and the retina was flat and well-attached. The patient maintained a prone position for three days post-surgery, followed by alternating between prone and right decubitus positions every 12 h. Subsequently, he received a total of five sessions of barrier PC in the left eye at 3 weeks, 1 month, 5 weeks, 1.5 months, and 2 months postoperatively.

At the 6-month follow-up, the retina remained flat and well attached, and the patient achieved a BCVA of 20/20 in the left eye without any complications (Figure 2C,D).

- Case 3

**Figure 3:**
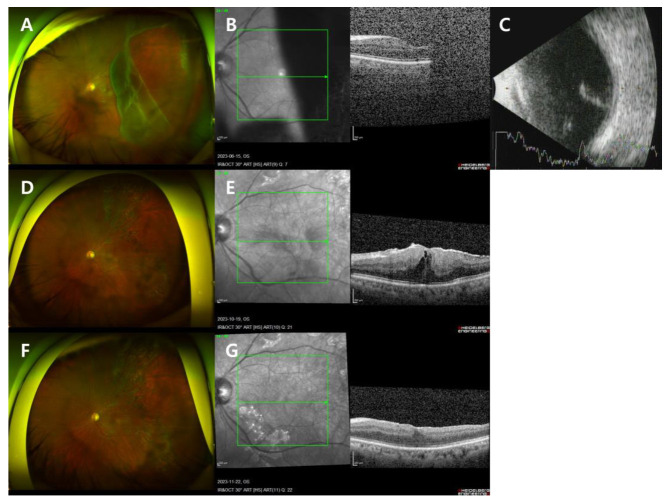
Case 3 patient’s left eye images. (**A**–**C**) On the day of presentation. (**A**) FP showed RRD with GRT in the superotemporal direction. (**B**) OCT showed macula-off RD. (**C**) B-scan showed RD. (**D**,**E**) Four months after the initial surgery. (**D**) FP showed a secondary epiretinal membrane (ERM). (**E**) OCT showed a secondary ERM. (**F**,**G**) Six months after the initial surgery. (**F**) FP showed a flat, well-attached retina. (**G**) OCT showed a flat, well-attached retina.

A 60-year-old man visited the clinic reporting floaters in the left eye that began 12 days prior and obscured vision that started the day before. He had a history of retinal tear in the right eye treated with barrier PC at a local ophthalmology clinic two years ago and had undergone cataract surgery in both eyes. There was no other medical history. Corrected visual acuity was 20/20 in the right eye and 20/100 in the left eye, with no significant findings in the anterior segment. FP, OCT, B-scan, and fundus examination revealed vitreous hemorrhage and GRT in the superotemporal direction with macula-off RRD in the left eye (Figure 3A–C). The axial length of the left eye was measured at 25.95 mm.

On the day of his visit, the patient underwent PPV, endolaser, fluid–air exchange, and intravitreal gas injection (C3F8 14%) in the left eye. The day after the surgery, his visual acuity in the left eye was hand motion, IOP was 11 mmHg, and the retina was well attached and flat. The patient maintained a prone position for three days post-surgery, followed by a right decubitus position.

Three months post-operation, the retina remained flat and well attached without complications, and the patient’s BCVA was 20/20. However, four months post-operation, a decrease in visual acuity in the left eye was observed along with the identification of a secondary epiretinal membrane (ERM). The patient underwent another surgery in the left eye, including PPV, endolaser, epiretinal membrane and internal limiting membrane peeling (ERM-ILMP), fluid–air exchange, and intravitreal gas injection (sulfur hexafluoride (SF6) 20%). Gas is not typically used in standard epiretinal membrane surgeries. However, this patient had RRD with GRT, and although the RRD with GRT was properly reattached, there remained a risk of retinal redetachment during manipulation. Given the patient’s high level of anxiety, and following a thorough discussion, we opted to use SF6 gas due to its shorter duration compared to C3F8 (Figure 3D,E).

Six months after the first surgery, the retina remained flat and free of ERM, and the patient’s BCVA was confirmed to be 20/20 without any complications (Figure 3F,G).

## 4. Discussion

In this study, all three cases were male, with an average age of 61 years. This is older than the average age of 42 years found in previous studies, but consistent with the higher male ratio [2,4]. All patients developed RRD with GRT without any specific trauma history. Among them, two had an axial length greater than the average of 22.6 ± 0.91 mm [18], with one case at 27.52 mm, significantly exceeding the criterion for high myopia set at 26 mm [19]. High myopia leads to retinal detachment by causing the thinning of the retina and vitreous degeneration, which increase the risk of retinal tears and breaks. Additionally, the elongated shape and increased axial length of the myopic eye create tractional forces and weak zones in the retina, further elevating the risk of detachment [19]. Two of the three cases were of idiopathic origin, and one was due to high myopia. Prior research indicates that approximately 54% of GRTs are idiopathic, and 25% are due to high myopia [1,2,5]. The variability in GRT presentations is further illustrated by recent case reports. Kohmoto et al. [6] reported two cases of GRT located in the intermediate periphery, highlighting the unusual positioning of tears in patients with spherically large eyeballs, which necessitates specialized surgical approaches. Samanta et al. [7] discussed a unique post-traumatic GRT case associated with a macular hole, emphasizing the role of trauma in complex retinal detachments and the need for tailored surgical interventions. Additionally, Sehgal et al. [8] described a rare case of GRT in a child with Marfan’s syndrome, underscoring the importance of considering genetic factors in the management of such cases. These reports, like ours, reinforce the necessity for individualized treatment plans based on the specific etiological and clinical characteristics of each GRT case. Patients presented with symptoms such as floaters, decreased vision, and obscured vision. Two visited within 10 days of symptom onset (Case 1: 5 days after symptom onset; Case 2: 1 day after symptom onset), and one after 12 days. Prior research indicates that two-thirds of GRT patients present within 10 days of experiencing visual symptoms [9]. In this study, two patients had macula-off RD. According to Ang, G.S. et al. [2], about half of GRT and RRD patients have macula-off RD.

We analyzed the characteristics of the general RRD with GRT patient population by reviewing multiple works from the literature. Cases from international publications on RRD with GRT have been organized in Table 1. Table 1 summarizes the demographic and clinical data from various studies on RRD with GRT patients. The youngest mean age reported is 35 years, with many cases occurring in patients in their 40s. This indicates that GRT should be considered and monitored even in relatively young patients. The younger age group likely experiences higher activity levels, which may contribute to the significant proportion of trauma-related GRT cases. Additionally, GRT occurs more frequently in male patients, highlighting the need for increased vigilance in this demographic. The primary etiologies for GRT include idiopathic causes and high myopia, both of which are challenging to prevent. This underscores the importance of rapid diagnosis and treatment as soon as GRT occurs. Trauma is one of the few preventable causes, emphasizing the need for precautions against trauma and falls. Surgical intervention results in a final retinal reattachment rate of at least 84.8%, indicating a relatively high success rate in reattachment. However, a BCVA of 20/40 or better after surgery indicates that the visual prognosis is often poor, highlighting the severity of this condition. Additionally, the occurrence of recurrent RD at relatively high rates suggests that RRD with GRT is a severe condition that requires careful management and monitoring.

Table 2 summarizes the characteristics and outcomes of the three patients in our study.

PPV is the preferred surgical treatment for GRT, involving techniques to correct the retinal flap, seal the retina, and provide tamponade with silicone oil or gas [9]. All patients in this study underwent PPV, endolaser photocoagulation, fluid–air exchange, and intravitreal gas injection (C3F8 14%). The use of perfluorocarbon liquids during surgery helps to flatten the retinal flap and facilitate reattachment. All three case patients achieved primary retinal reattachments. According to prior research, RRD with GRT has an 88% primary reattachment rate and a 95% final reattachment rate [2]. In addition to anatomical outcomes, functional outcomes should also be considered, with visual acuity being the most representative measure. The British giant retinal tear epidemiology eye study (BGEES) study reported 42% of patients achieving a BCVA of 20/40 or better [2]. In this study, all three cases showed a visual acuity of 20/40 or better after approximately 6 months post-surgery, with all three achieving a BCVA of 20/20 (Table 1 and Table 2). Moreover, previous studies indicate that recurrent RD can occur in approximately 11% [14] to as many as 50% [20] of cases, depending on the research. In this study, none of the patients experienced recurrent RD during the six-month follow-up period. Considering that most recurrences (69%) occur within six months after surgery [22], this is considered an acceptable outcome. Nonetheless, since late recurrences can occur from 1.1 years to 10.4 years post-surgery [23], it is important to remain vigilant about the potential for recurrent RD in these patients. Other possible complications following surgery for GRT include proliferative vitreoretinopathy (PVR), cataract formation, secondary glaucoma, macular hole formation, and secondary ERM [2]. However, aside from the secondary ERM that developed in Case 3 four months after the first surgery, none of the patients experienced any of these complications.

Factors contributing to the favorable anatomical outcomes and functional outcomes for RRD with GRT patients may include the following:

**Rapid surgical intervention:** All three were operated on within four days of their visits, with two undergoing surgery on the days of their visits (Cases 2 and 3). Considering that the mean preoperative time for GRT surgery was reported as 1.8 weeks in a multicenter study [24], the prompt application of surgical treatment upon admission in this study likely had a positive impact. Unsurprisingly, according to the study by Guner, M.E. et al. [25], early intervention positively affected functional outcome. Additionally, Angermann et al. [26] found that patients who received surgery within 24 h of diagnosis had significantly better visual outcomes than those who underwent surgery more than 72 h after diagnosis. These studies have shown that immediate surgical intervention for RRD with GRT leads to better visual acuity and higher retinal reattachment rates compared to delayed surgeries. Further supporting this, Frings et al. [27] demonstrated that visual recovery after macula-off RD is significantly better when surgery is performed within the first 72 h compared to later interventions. Additionally, Van Bussel et al. [28] and Greven, M. A. et al. [29] reported that the duration of macula-off retinal detachment has a substantial impact on long-term visual outcomes, emphasizing the need for timely surgical intervention.

Early surgical intervention is critical in preventing further complications and enhancing the chances of visual recovery. The importance of minimizing the time between diagnosis and surgery cannot be overstated in managing RRD with GRT cases effectively.

**Adequate surgical treatment:** We ensured that all patients had confirmed posterior vitreous detachment before proceeding with the surgery. Perfluorocarbon liquid was used in all cases. Due to the large and flapping giant retinal tear, it was necessary to flatten it with perfluorocarbon liquid. The flap was flattened down using perfluorocarbon liquid, and a partial retinectomy was performed on the rolled-up margin of the flap. After the flap was properly flattened, the perfluorocarbon liquid was completely removed during the fluid–air exchange process, ensuring that it did not enter the subretinal space and cause further complications. Intraoperative laser treatment was performed thoroughly as usual.

While some surgeons prefer using silicone oil or a scleral buckle during GRT procedures, we opted for C3F8 gas to achieve perfect retinal adhesion. The choice of C3F8 gas was made to avoid the disadvantage of needing to perform an additional surgery to remove the silicone oil later, which can be inconvenient and pose additional risks for the patient. C3F8 gas provides a long-lasting tamponade effect, which is beneficial for the healing process and maintaining retinal attachment.

Additionally, in this study, there were no intraoperative complications, such as tear slippage. Tear slippage can occur in up to 16% of cases [30]. Tear slippage can greatly affect the success of the surgery and the patient’s recovery, so avoiding this and other intraoperative complications is crucial for achieving a better outcome for RRD with GRT patients.

**Postoperative inflammation control:** Preoperatively, patients were instructed to use bromfenac eye drops twice a day (bid) until surgery. Postoperatively, all patients received intramuscular dexamethasone and oral prednisolone along with antibiotics, contributing to effective inflammation control. Patients were prescribed oral prednisolone for 6 days. The dosage of oral prednisolone was tapered from 30 mg for 2 days, to 20 mg for 2 days, and finally to 10 mg for 2 days. Post-surgery, for a certain period, patients were administered moxifloxacin eye drops four times a day (qid), prednisolone eye drops qid, bromfenac eye drops twice a day (bid), and ofloxacin ointment at bedtime to aid in inflammation control and recovery. Postoperative inflammation increases the risk of complications, such as PVR, after RRD surgery, potentially leading to surgical failure [31]. Several previous studies have mentioned that effective postoperative inflammation control leads to favorable outcomes after surgery. Aptel, F. et al. [31] stated that topical anti-inflammatory therapy during the perioperative period is beneficial not only for patients undergoing uncomplicated cataract surgery but also after vitrectomy. According to Kim, S.J. et al. [32], using topical ketorolac, a nonsteroidal anti-inflammatory drug (NSAID), in the perioperative period was associated with improvements in inflammation and visual outcomes in vitreoretinal surgery. The use of oral corticosteroids lacks solid consensus, but studies, including a large randomized clinical trial by Koener, F. et al. [33], suggest that oral prednisone after surgery can reduce the incidence of postoperative grade B PVR [33,34]. PVR is particularly significant as it often necessitates multiple surgeries and is associated with poorer visual outcomes. In addition, inflammation-associated retinal complications are well noted in the literature. For instance, a study by Nguyen, D.D. et al. [35] demonstrated the effectiveness of retina-permeating and long-acting nanotherapeutics, such as resveratrol and metformin, in reducing macular degeneration by controlling inflammation. Therefore, effective postoperative inflammation control can be a contributor to favorable outcomes in RRD with GRT patients.

**Removal of vision-reducing factors:** The common postoperative complications following surgical interventions for RRD with GRT include cataract formation, ERM, macular holes, posterior capsule opacification, PVR, and retinal redetachment [2]. These complications are the primary reasons for reduced vision after surgery for RRD with GRT. Factors contributing to these postoperative complications include the extent and severity of retinal tear, preoperative visual acuity, the presence of PVR, and the timing of surgical intervention. Worse presenting visual acuity, larger extents of retinal tear (≥150 degrees), and the presence of macula-off status are associated with poorer outcomes and higher complication rates. As noted by Ting et al. [36], these factors significantly affect both functional and anatomical success in GRT-related RRD cases.

While it is not possible to reduce the size of an already-enlarged retinal detachment, ensuring a patient remains as stable and free from impact as possible before surgery, including the use of a wheelchair, and performing rapid surgical intervention can prevent the further enlargement of retinal tear. As mentioned above, we also aimed to remove vision-reducing factors by controlling inflammation to prevent PVR. Additionally, cataract formation and ERM are major complications that can negatively impact visual outcomes. Two patients had already undergone cataract surgery, and for the one case (Case 1) who had not, cataract surgery was performed along with PPV to prevent the development of cataracts. In Case 3, a secondary ERM developed four months after the initial surgery, leading to a decrease in recovering vision. However, after ERM removal surgery, an excellent BCVA of 20/20 was ultimately achieved. Therefore, favorable outcomes for RRD with GRT patients can be achieved by the timely removal of vision-reducing factors that develop postoperatively.

**Frequent follow-ups and additional barrier photocoagulation:** All patients had a follow-up the morning after their surgery, with an additional one–two follow-ups occurring within the first week. For two patients, these weekly follow-ups continued for up to a month post-surgery. Frequent follow-ups enabled the prompt correction of any incorrect post-op positions and close monitoring of eye drop compliance. Moreover, as mentioned above, retinal redetachment is one of the most common postoperative complications [2]. Early detection and intervention during these follow-ups are essential in managing and preventing any emerging issues. Although appropriate intraoperative laser treatment was performed during the surgery, the tear was large and the risk of redetachment was high, so additional barrier laser treatments were cautiously applied only around the tear margins. It is speculated that performing additional barrier PC as the gas was dissipating in two of the patients might have played a role in preventing retinal redetachment.

This study’s limitations are notable and warrant discussion. First, despite the rarity of RRD with GRT, the number of cases studied is still significantly insufficient to draw definitive conclusions. A larger sample size would provide a more robust dataset for statistical analysis, allowing for a better understanding of the variability in outcomes and potential complications. Additionally, the small sample size limits the generalizability of our findings to the broader population of patients with RRD and GRT. Second, all three cases were managed at the same hospital by the same surgeon. Our hospital and surgeon may receive referrals for more complicated cases due to their specialized expertise, potentially leading to a different patient population than what might be seen at other locations. This concentration of complex cases could skew the results, as the outcomes observed may not be reflective of those in a more generalized setting. Third, the results are based on a follow-up period of up to 6 months, which may not be sufficient to capture the long-term efficacy and potential complications of the treatment. According to the study by Goezinne, F. et al. [9], about 30% of cases result in redetachment when observed for more than a year, highlighting the importance of longer follow-up durations. A short follow-up period may fail to detect late-onset complications or recurrences, which are crucial for assessing the durability of surgical outcomes. Extending the follow-up period in future studies would provide more comprehensive data on the long-term success rates.

## 5. Conclusions

In this study, we evaluated three cases of RRD with GRT treated at CHA Bundang Medical Center, highlighting the condition’s clinical features, treatment approaches, and outcomes. Given the small sample size, it is not meaningful to discuss whether our results indicate success. However, after extensively reviewing the literature and reflecting on our case experiences, we propose several key factors that may contribute to better outcomes for RRD with GRT patients: rapid surgical intervention, adequate surgical treatment, effective postoperative inflammation control, frequent post-op follow-ups, and proactive management of potential vision-reducing complications. These factors can improve anatomical and functional results and serve as a model for treating RRD with GRT. A comprehensive and meticulous treatment protocol can lead to better outcomes in managing complex retinal detachments associated with giant retinal tears. Future studies with larger cohorts and extended follow-ups are essential to validate our findings and refine treatment protocols.

## Figures and Tables

**Table 1:** Demographic and clinical data of rhegmatogenous retinal detachment with giant retinal tear patients.

Author (Year)	Eyes (n)	Mean Age (Years)	Sex (%)		Etiology			Retinal Reattachment		BCVA 20/40 or Better after OP	Recurrent RD
			M	F	Idiopathic	Trauma	High Myopia	Primary	Final		
Kertes et al. [20] (1997)	162	N/A	N/A	N/A	N/A	41 (23.5%)	20 (12.3%)	78 (48.1%)	147 (90.7%)	24 (14.8%)	80 (49.4%)
Ambresin et al. [14] (2003)	18	44	72	28	N/A	2 (11.1%)	7 (38.9%)	16 (88.9%)	17 (94.4%)	9 (50.0%)	2 (11.1%)
Gosh et al. [3] (2004)	29	35	86	14	10 (34.5%)	9 (31.0%)	10 (34.5%)	19 (65.5%)	25 (86.2%)	N/A	6 (20.7%)
Sirimaharaj et al. [13] (2005)	62	44	84	16	N/A	19 (30.6%)	10 (16.1%)	49 (79.0%)	58 (93.5%)	27 (43.5%)	13 (21.0%)
Goezinne et al. [9] (2008)	30	53	N/A	N/A	N/A	4 (13.3%)	N/A	21 (70.0%)	29 (96.7%)	N/A	9 (30.0%)
Lee et al. [21] (2008)	128	40	91	9	N/A	17 (13.3%)	52 (40.6%)	71 (71.7%)	84 (84.8%)	N/A	15 (15.2%)
Ang et al. [2] (2010)	62	42	72	28	34 (54.8%)	10 (16.1%)	11 (17.7%)	50 (87.7%)	54 (94.7%)	24 (42.1%)	12 (21.1%)

OP = operation; BCVA = best-corrected visual acuity; and RD = retinal detachment.

**Table 2:** Comparison of the age, sex, axial length, initial visual acuity, post visual acuity, time taken to the procedure, additional barrier PC, and complications between different cases.

	Age	Sex	AXL (mm)	Days to OP	Follow-Up (Months)	BCVA before OP	BCVA after OP	Additional Barrier PC	Complication
Case 1	57	M	27.52	4	6	20/100	20/20	3	-
Case 2	66	M	23.91	0	6	20/63	20/20	5	-
Case 3	60	M	25.98	0	6	20/100	20/20	0	Secondary ERM

M = male; OP = operation; AXL = axial length; BCVA = best-corrected visual acuity; barrier PC = barrier photocoagulation; and ERM = epiretinal membrane.

## Data Availability

The original contributions presented in the study are included in the article; further inquiries can be directed to the corresponding author.
